# Special care dentistry undergraduate curriculum in Asia-Pacific: a descriptive study of 31 dental schools

**DOI:** 10.3389/froh.2026.1855519

**Published:** 2026-07-07

**Authors:** Gabriel Keng Yan Lee, Guang Xu David Lim, Aminda Faizura Omar, Mathew Albert Wei Ting Lim

**Affiliations:** 1 Faculty of Dentistry, National University of Singapore, Singapore, Singapore; 2 Dental School, University of Western Australia, Nedlands, Western Australia, Australia; 3 Special Care Dentistry Unit, Faculty of Dentistry, Universiti Teknologi MARA, Sungai Buloh, Selangor, Malaysia; 4 Melbourne Dental School, University of Melbourne, Carlton, Victoria, Australia

**Keywords:** special care dentistry, Asia-Pacific, dental education, learning outcomes, undergraduate

## Abstract

**Background:**

Special care dentistry (SCD) addresses the oral health needs of individuals whose disabilities, medical complexities, or social circumstances limit access to dental care. With rapid population ageing and a high disability prevalence, the Asia-Pacific region faces a growing demand for dentists competent in managing patients requiring special care. For the majority of patients requiring special care, routine basic dental care is helmed by the general dentist. While the International Association for Disability and Oral Health (iADH) has published undergraduate curriculum guidance for SCD, its implementation and attainment of learning objectives within dental schools in this region has not been examined.

**Aim:**

This study aimed to describe the status of undergraduate SCD education across dental schools in Asia-Pacific, using the iADH guidance as a benchmark, and to identify strengths and gaps to inform future curriculum development.

**Methods:**

A descriptive survey was conducted among faculty members involved in undergraduate SCD teaching in dental schools in the Asia-Pacific region. An online questionnaire was administered between December 2025 to February 2026, assessing the extent to which programmes fulfilled learning outcomes across the 6 competency domains of the iADH guidance. Data on course structure, teaching hours, educational modalities, and perceived limitations were collected. Quantitative data was analysed using descriptive statistics, while open-ended responses were analysed thematically.

**Results:**

Thirty-one dental schools from 11 countries and jurisdictions participated. SCD was more commonly delivered as an independent subject, with a median of 15 h of didactic teaching and 8 h of clinical exposure. Overall, more schools reported sufficient fulfilment of the learning outcomes, although only three fully adopted the guidance. Common challenges included limited clinical exposure, shortages of trained academic staff, and considerations in adapting the guidance to local contexts.

**Conclusion:**

Undergraduate SCD education in the Asia-Pacific region is active but variable within the sample institutions. Given uneven regional representation, the findings of this study serve as useful exploratory data. While the iADH guidance provides a valuable framework, greater flexibility, contextual relevance, and support for clinical training and educational leadership should be advocated to ensure graduates are practice-ready to meet the oral health needs of patients requiring special care.

## Introduction

The impact of oral health on an individual's general health and quality of life is evident. Calls to develop and strengthen oral healthcare services to reduce health inequities have been underway in recent years (1). Notably, oral diseases tend to be more prevalent in groups with special care needs. These include persons with disabilities, marginalised individuals, and adults with medical complexities (2). Moreover, many countries are experiencing rapidly ageing trajectories, increasing the disease burden and complexity of profiles faced by dental professionals (3).

Special care dentistry (SCD) is described as dentistry for “*those with a disability or activity restriction which directly or indirectly affects their oral health*” (4). For many countries, SCD starts with philanthropic or charitable intent which gradually becomes consolidated as professional societies or associations, for example the Japan Society for Disability and Oral Health's formation in 1973 (2, 5). This later progresses with formal inclusion into dental curricula, directorate recognition as an official dental specialty, and subsequently normalised within public policy to tackle oral health inequity (6, 7).

To ensure that the workforce is prepared to manage these sub-populations, dental schools around the world are encouraged to incorporate SCD, or elements of it, within undergraduate dental degrees (i.e., basic qualification required for licensure) (8). Educational interventions include didactics, extramural teaching, clinical observerships, and clinical rotations into their curriculum centred on the special care patient (9). The aim of this is to ensure a baseline level of competency of the dental profession in the management of persons with special care needs, thereby reducing oral health inequalities and inequities.

In 2012, the International Association for Disability and Oral Health (iADH) set out a guidance document for SCD curriculum at undergraduate level, developed through an international Delphi process (10, 11). The guidance sets out standards for learning outcomes (i.e., knowledge, skills, and attitudes) through six competency areas in SCD (12):

- Scope of special care dentistry,
- Access and barriers to oral health for people with disability & other marginalised groups,
- Consent for people requiring special care,
- Communication skills in special care dentistry,
- Impact of impairments, disabilties & systemic conditions on oral health & oral function, and
- Clinical management of patients requiring special care dentistry.

Home to around 57% of the world's population, the Asia-Pacific region is experiencing the world's fastest ageing population, with an estimated 1.3 billion aged 60 and above by 2050 (13). Furthermore, an estimated 1 in 6 people live with a disability (14). To meet the increasing oral healthcare burden of these populations, the region has rapidly developed SCD services over the last two decades, with specialty recognition formalised at the national level including Australia (since 2003), New Zealand (2003), Malaysia (2012), Japan (2017), Singapore (2025), and Thailand (2025) (7, 15). Distinguishing SCD as a specialised field of dentistry at the national level can signal governmental recognition of the patient population, often those with special healthcare needs and geriatric conditions, that requires a specific skillset and/or resources (16). This may also influence delineation of service delivery across various levels of the healthcare system, including community-based primary dental care services. Furthermore, specialty-status calls for advanced domain expertise, advocacy and advice to policymakers, and educational leadership for the future generations of dental practitioners (17).

Notably, persons requiring special care do not always require specialist-level services, such as those with well-controlled comorbidities or well-acclimatised behavioural profiles. These individuals can seek care with general dentists, who should ideally make reasonable adjustments to accommodate their needs and ensure equity (18). Moreover, general dentists form the majority of the oral health workforce. In 2025, the Commission on Dental Accreditation (CODA) in the United States reviewed its predoctoral education standards to assert that “*graduates **must** be competent in assessing and managing the treatment of patients with special needs*” (19). This highlights the importance of ensuring that the training of general dentists can adequately meet basic professional competencies in the care for SCD patients. In a recently published survey, about 83% of dental schools in the UK and Ireland applied the iADH guidance to their undergraduate special care dentistry teaching (20). The study also identified strengths and gaps in the dental schools surveyed, including lack of clinical exposure and support from senior management to teach SCD. However, the implementation and achievement of learning outcomes based on this guidance has not been well studied in the Asia-Pacific region.

Hence it is imperative to understand the level of training provided to undergraduate dental students in the region to ensure the oral healthcare system is able to meet the patient with special care needs at all levels of complexity. In order to ensure the oral healthcare system is able to meet the patient with special care needs at all levels of complexity, it is imperative to evaluate the immediate response (Kirkpatrick's level 1 - reaction) to the additional demands in this curriculum, and how well the learning can be imparted (Kirkpatrick's level 2 - learning) (21). The aim of this study is therefore to report on the status of SCD education in dental schools across the Asia-Pacific, using the iADH undergraduate guidance as a yardstick, and to identify areas of strength and gaps in the guidance that can be considered for review.

## Methodology

A descriptive survey of dental schools across Asia-Pacific was undertaken. Eligible participants were faculty members, either part-time or full-time, involved in the teaching of SCD in a dental school situated in the Asia-Pacific region. There were no limitations to the number of respondents per country. Only one response per institution was allowed. To note, the Asia-Pacific region comprises a large and heterogeneous number of dental schools, with substantial variation in distribution across countries. As such, the present sample represents a subset of institutions rather than a comprehensive regional census. The survey form was available online from December 2025 to February 2026 on a Qualtrics platform *(Qualtrics LLC, 2025).* Convenience and snowball sampling were employed, including sharing the survey link through special care dentistry organisations with connection to the iADH. A participant information page was provided before the survey, and informed consent was implied through voluntary participation.

The survey form was developed by three authors (GKYL, GXDL, MAL) involved in undergraduate SCD teaching in their respective dental schools. Additionally, three authors (GKYL, AFO, MAL) were members of the iADH education committee that oversaw the review of the undergraduate guidance at the time of writing. The survey form was developed directly from the competency statements of each of the 6 domains of the iADH guidance, where respondents were asked to score on a 5-point Likert scale the extent of which their undergraduate programmes fulfilled (not taught at all; barely taught/largely not fulfilled; taught but insufficiently fulfilled; mostly fulfilled but with notable gaps; taught and sufficiently fulfilled). Additionally, respondents also provided information on the general structure of the SCD teaching (embedded, independent module, or not taught at all), the number of didactic and clinical hours, and the educational methodologies used (e.g., blended learning, role-play, simulations). Open-ended questions sought direct feedback on the iADH guidance (e.g.,“*What domains or learning objectives stated above do you feel should not be required in undergraduate teaching”),* any additional domains and learning objectives taught in the respondent's university, and any limitations faced in being able to teach SCD as guided by the iADH guidance.

Descriptive statistics were used to summarise the baseline characteristics of the survey data. Domain scores were calculated as the mean score for each domain and are presented as median with 25th and 75th percentiles. Radar plots were generated to visually compare the coverage of the six learning objective domains across country groupings. Differences in domain scores between independent groups were assessed using the Kruskal–Wallis test for variables with more than two groups and the Mann–Whitney U test for two-group comparisons. For significant Kruskal–Wallis tests, *post-hoc* pairwise comparisons were performed using Mann–Whitney U tests with Bonferroni correction for multiple comparisons to reduce the risk of Type I error due to multiple testing. Statistical significance was set at *p* < 0.05. All analyses and visualisations were performed using R software version 4.3.1. Qualitative responses were analysed using an abductive, reflexive thematic analysis approach. Two researchers (GKYL, AFO) independently coded the data, whereby codes and emergent themes were compared and refined through iterative discussion to achieve consensus. Thereafter, the finalised themes were discussed and agreed upon between all four researchers.

The study was approved by the National University of Singapore Institution Review Board via exemption (NUS-IRB-2025-1017).

## Results

**Figure 1:**
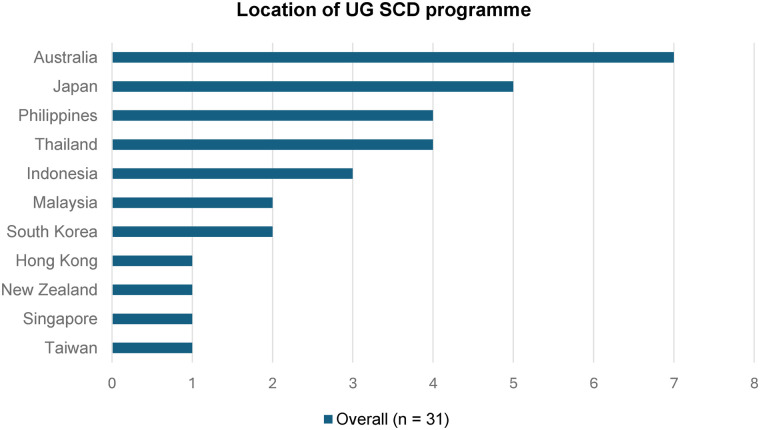
Distribution of respondents.

Participants representing 31 dental schools across 11 countries and jurisdictions responded to the survey (Figure 1). Regionally, East Asia was represented by nine dental schools, Southeast Asia by fourteen, and the Pacific by eight. There were no responses from North, Central, West, or South Asia. As such, regional representation was uneven.

SCD was taught as an independent subject in 19 schools (59.4%), while 8 schools taught it as part of other disciplines (25.0%) (Table 1). The remainder taught SCD as both an independent subject, but had components also embedded within other subjects. SCD was taught typically around 15.0 h (didactics) and 7.5 h for clinical practice. Lectures were the most common method of delivery, followed by observerships and case-based learning.

**Table 1:** Descriptive statistics of SCD teaching and course design.

Details of responses	Overall (*N* = 31)
Academic seniority (of faculty staff overseeing SCD)	
Professor	4 (12.9%)
Associate professor	9 (29.0%)
Assistant professor	7 (22.6%)
Senior lecturer	5 (16.1%)
Lecturer/tutor	5 (16.1%)
Others	1 (3.2%)
Independent vs. embedded	
Independent subject	18 (58.1%)
Embedded	8 (25.8%)
Independent, with some components embedded	5 (16.1%)
Year taught	
4 years	7 (22.6%)
5 years	8 (25.8%)
6 years	13 (41.9%)
Others	3 (9.7%)
Teaching hours (diadactic)	
Median (Q1, Q3)	15.00 (12.00, 34.00)
Teaching hours (clinical)	
Median (Q1, Q3)	8.00 (3.00, 24.00)
Delivery methods	
Lectures	30 (96.8%)
Case based learning	24 (77.4%)
Obseverships	24 (77.4%)
Seminars	18 (58.1%)
Hands on	16 (51.6%)
Blended learning	8 (25.8%)
Role play	6 (19.4%)
Others	3 (9.7%)
Broad topics covered	
Developmental disabilities	31 (100.0%)
Medically complex	30 (96.8%)
Physical disabilities	29 (93.5%)
Physical conditions	27 (87.1%)
Geriatrics	25 (80.6%)
Homelessness	10 (32.3%)

**Figure 2:**
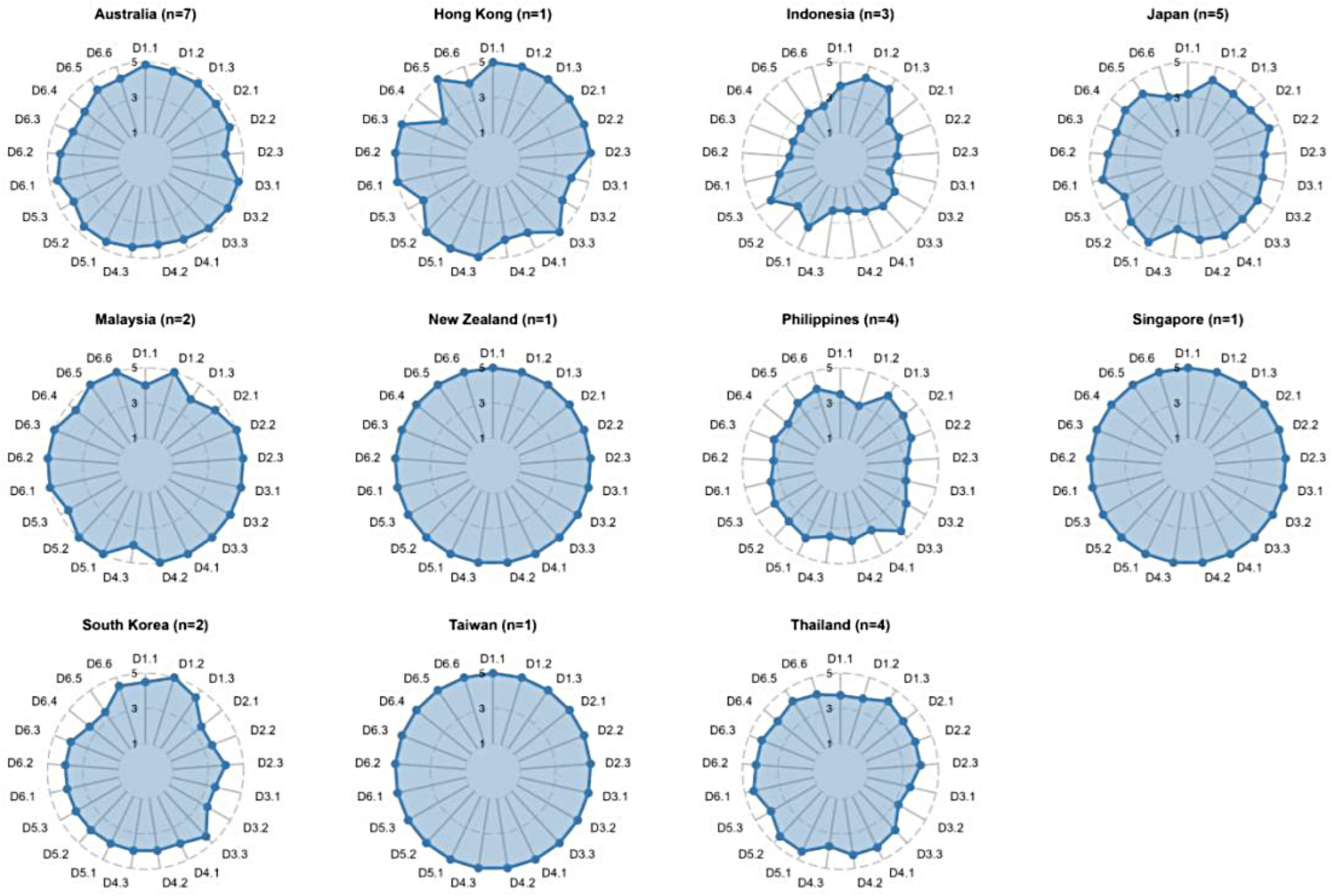
Attainment of learning objectives* across each country/jurisdiction. *D1.1 refers to Domain 1 Statement 1 of the iADH undergraduate guidance (12) (i.e., “*Describe the cultural, legal and social context of people with disability and other marginalised groups*.”), etc.

**Figure 3:**
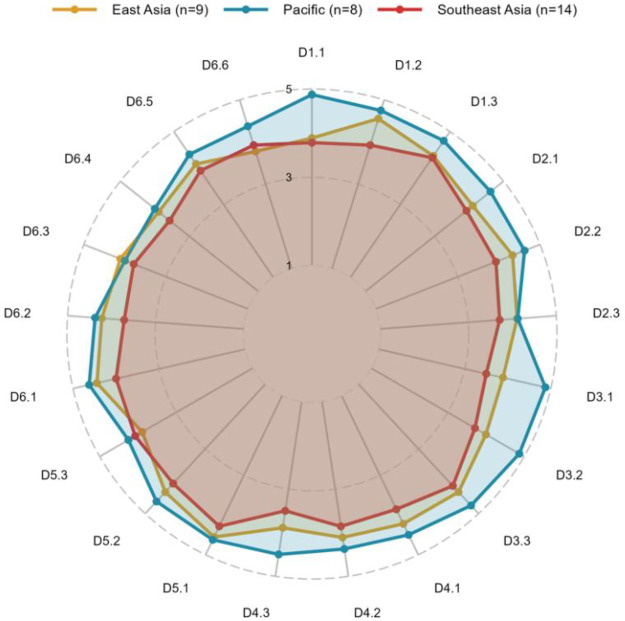
Attainment of learning objectives categorised by region.

**Figure 4:**
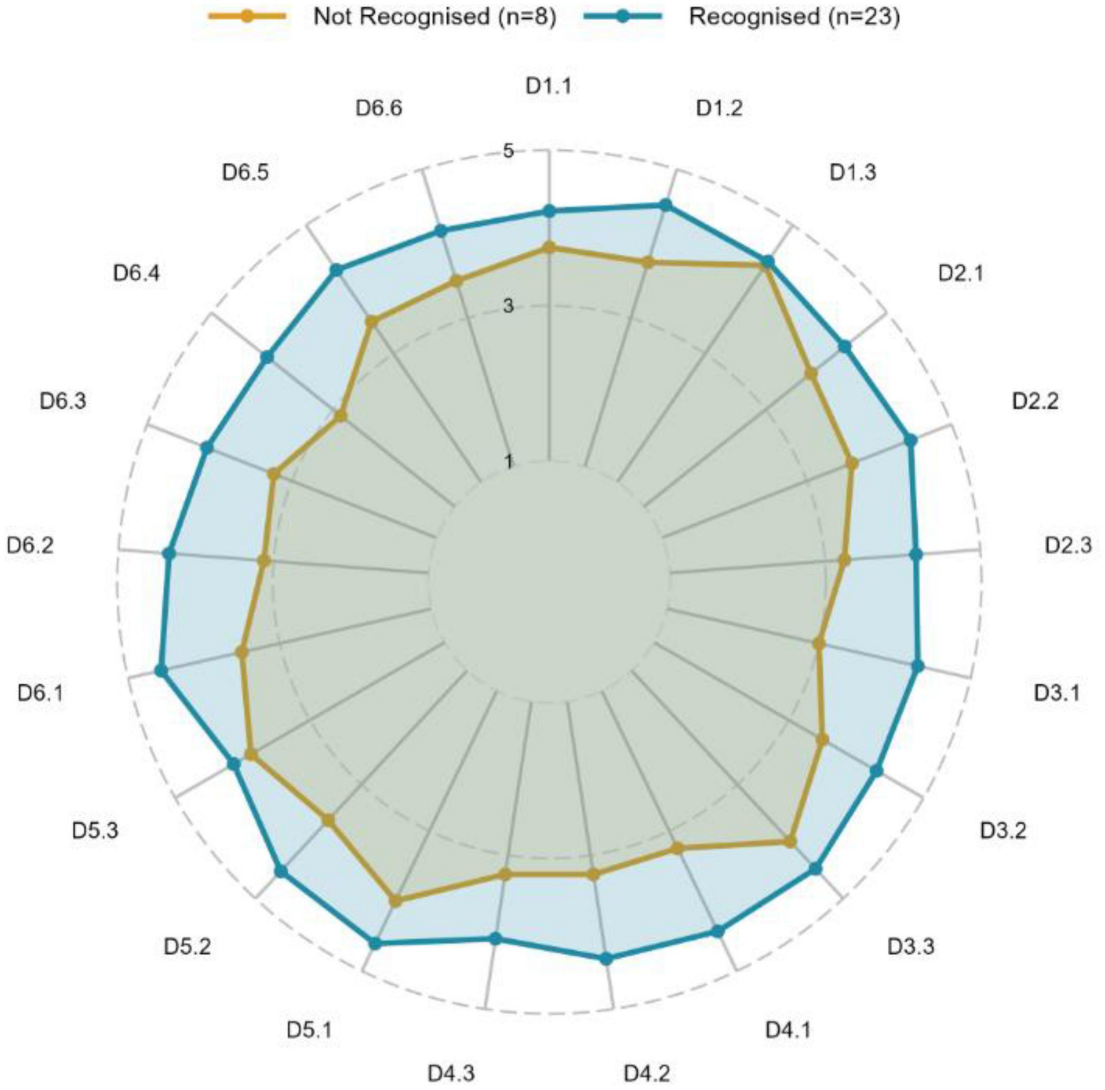
Attainment of learning objectives when categorised into countries/jurisdictions with national specialty recognition of SCD and those without.

**Figure 5:**
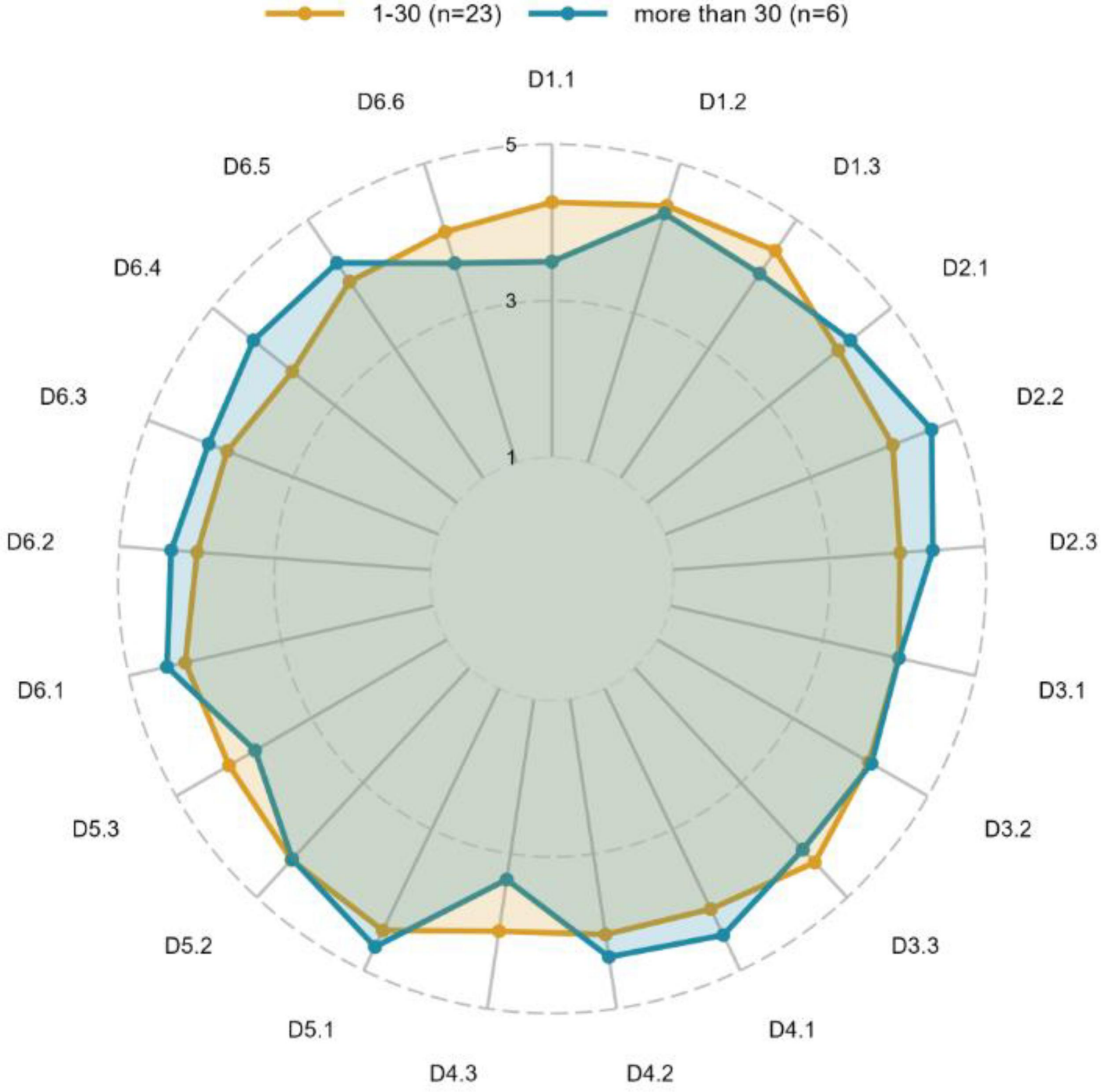
Attainment of learning objectives when SCD is allocated more or less than 30 h of clinical practice.

**Figure 6:**
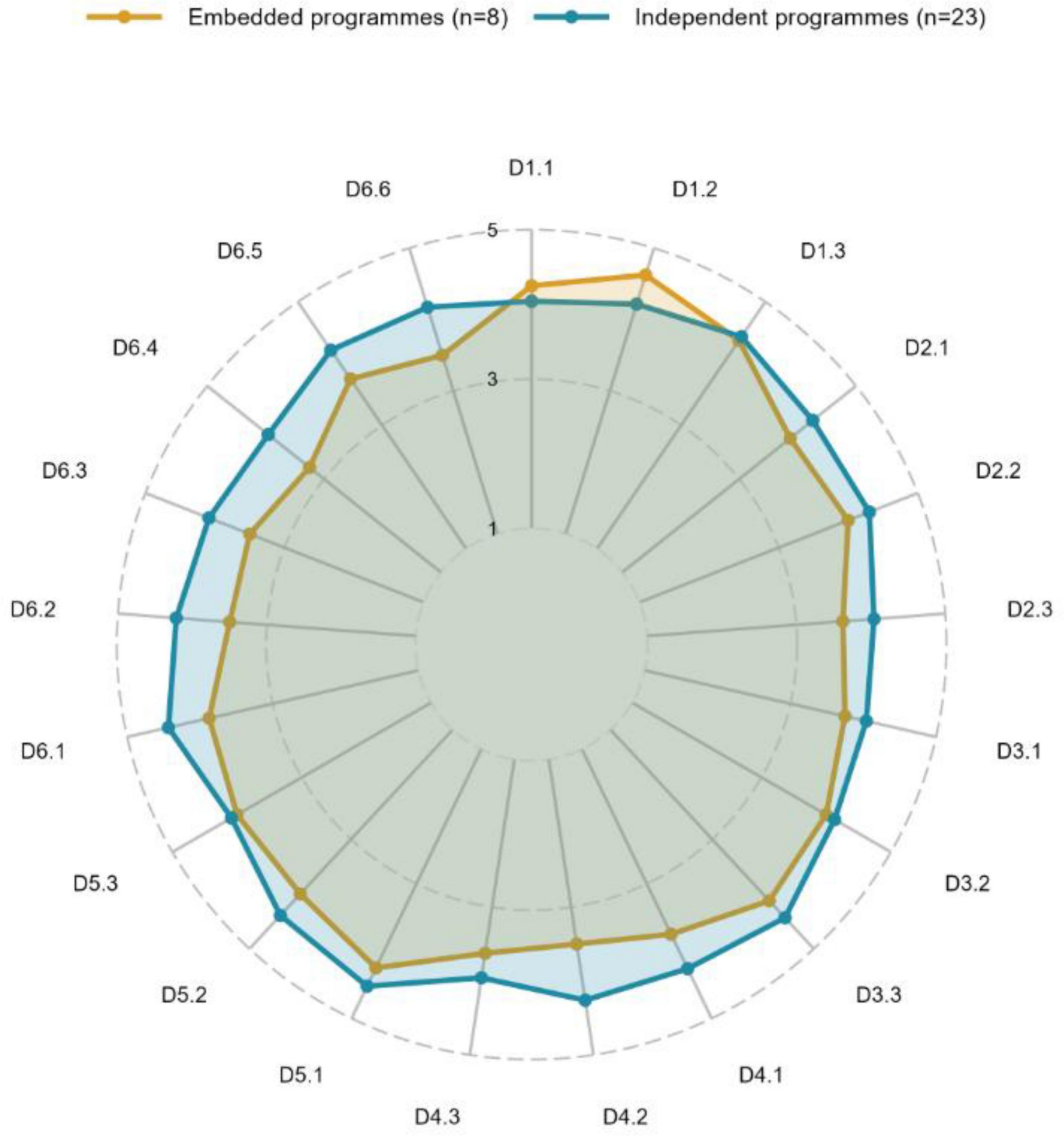
Attainment of learning objectives when SCD is embedded or an independent programme.

Each country/jurisdiction is represented by radar plots of the median scores of each learning outcome statement per domain (Figure 2). The higher the median score of each learning outcome, the greater the distance from the centre of the plot. Overall, 3 dental schools reported to be fully adopting the curriculum guidelines, while the rest reported variations in the extent of implementation. When categorised by region, dental schools in the Pacific region appear to most substantially fulfil the learning outcomes across the iADH guidance compared to East Asia and Southeast Asia dental schools (Figure 3). Countries/ jurisdictions with SCD recognised as a specialty at the national level surpassed those without specialty recognition in the fulfillment of learning outcomes (Figure 4), apart from statement 1C (“Demonstrate positive attitudes in relation to human difference and diversity”). The number of clinical hours (Figure 5) and didactic hours dedicated to SCD teaching, and whether it was taught as an independent subject or embedded in other subjects (Figure 6) did not appear to have a significant impact on fulfilling the learning outcomes (Table 2).

**Table 2:** Differences in domains achieved when comparing between course features.

Features of undergraduate course	Group	Scope of SCD (Domain 1)	*P*-value	Access & barriers (Domain 2)	*P*-value	Patient consent (Domain 3)	*P*-value	Communication skills (Domain 4)	*P*-value	Impact of impairments (Domain 5)	*P*-value	Clinical management (Domain 6)	*P*-value
Region	East Asia (*n* = 9)	4.3 (3.7, 5.0)	0.025*	4.0 (4.0, 5.0)	0.561	4.3 (3.3, 4.7)	0.023*	4.0 (4.0, 4.3)	0.354	4.0 (4.0, 5.0)	0.527	4.0 (3.7, 4.5)	0.683
	Oceania (*n* = 8)	5.0 (4.9, 5.0)		4.7 (3.9, 5.0)		5.0 (4.9, 5.0)		4.5 (4.0, 5.0)		4.8 (4.4, 5.0)		4.6 (3.8, 4.8)	
	Southeast Asia (*n* = 14)	4.3 (3.4, 4.7)		4.0 (2.8, 4.9)		4.0 (3.1, 4.6)		4.0 (2.9, 4.7)		4.2 (4.0, 4.7)		4.0 (3.2, 4.8)	
SCD specialisation recognised	Not Recognised (*n* = 8)	4.3 (3.2, 4.7)	0.194	3.7 (2.6, 4.2)	0.061	3.8 (3.2, 4.3)	0.029*	3.3 (2.5, 4.3)	0.073	4.0 (3.6, 4.4)	0.119	3.2 (2.5, 3.8)	0.021*
	Recognised (*n* = 23)	4.7 (3.8, 5.0)		4.7 (4.0, 5.0)		4.7 (4.0, 5.0)		4.0 (4.0, 5.0)		4.7 (4.0, 5.0)		4.5 (3.8, 4.8)	
Clinical hours	1 to 30 (*n* = 23)	4.7 (4.2, 5.0)	0.167	4.0 (3.5, 5.0)	0.488	4.3 (3.8, 5.0)	0.740	4.0 (3.8, 5.0)	0.640	4.7 (4.0, 5.0)	1.000	4.0 (3.4, 4.8)	0.588
	more than 30 (*n* = 6)	3.8 (3.7, 4.2)		4.3 (4.0, 4.9)		4.2 (3.5, 4.8)		4.0 (3.8, 4.5)		4.5 (3.8, 5.0)		4.2 (3.8, 4.9)	
Didactic hours	1 to 30 (*n* = 21)	4.7 (4.0, 5.0)	0.391	4.7 (4.0, 5.0)	0.725	4.3 (3.7, 5.0)	0.406	4.3 (3.7, 5.0)	0.921	4.3 (4.0, 5.0)	0.613	4.3 (3.5, 4.8)	0.902
	more than 30 (*n* = 8)	5.0 (3.9, 5.0)		4.3 (3.9, 4.8)		4.8 (4.1, 5.0)		4.0 (3.9, 4.5)		4.7 (4.4, 5.0)		4.2 (3.8, 4.5)	
Programme structure	Embedded programmes (*n* = 8)	4.7 (4.2, 5.0)	0.626	3.8 (3.3, 4.8)	0.331	4.2 (3.8, 5.0)	0.889	4.2 (3.2, 4.4)	0.462	4.5 (3.8, 4.7)	0.471	3.6 (2.8, 4.6)	0.123
	Independent programmes (*n* = 23)	4.3 (3.7, 5.0)		4.7 (4.0, 5.0)		4.3 (3.5, 5.0)		4.0 (3.8, 5.0)		4.7 (4.0, 5.0)		4.3 (3.8, 4.9)	

Data presented as Median (25th, 75th percentile). *p*-values calculated via Kruskal–Wallis test and Mann–Whitney U test.

a Pactific region statistically higher than East Asia in Domains 1 and 3, based on Bonferroni pairwise comparisons.

From the thematic analysis of the open-ended responses, majority of the respondents felt the guideline was adequate but did have opinions on ways to make it more applicable today.

“Everything is fine, it's a matter of placing and phasing of each object and course” (Southeast Asia respondent)

“The domains are well structured in terms of general requirements in an ideal scenario; however, they leave room for broad interpretation” (Pacific respondent)

Several felt that the guidance lacked adaptability specifically to the incorporation of local issues.

“For greater regional relevance, consider adaptability for integration of local disease patterns and public health needs as well as adding objectives pertaining to teledentistry and digital literacy.” (Southeast Asia respondent)

“It needs revision to include some region-based topics to cater to the growing disabled population.” (Southeast Asia respondent)

When identifying limitations in their dental schools in teaching SCD effectively, respondents frequently highlighted a shortage of academic staff available to teach the subject, and the lack of clinical hours dedicated for the management of patients with special care needs.

“One of the primary limitations.. is the shortage of academic staff who are strongly motivated and specifically committed to SCD education.” (East Asia respondent)

As one participant (East Asia respondent) opined that there was a lack of awareness of the importance of SCD, “.. Special Care Dentistry is often perceived as a secondary or peripheral area, rather than a core discipline requiring dedicated educational leadership”

On the matter of guideline suggestions, some respondents suggested the inclusion of lesson plans or samples of how curricula are executed for better preparedness and mentioned by these respondents:

“Would be helpful to include teaching curriculum samples and lesson plans as well.” (Southeast Asia respondent)

Other suggestions included integration of interprofessional collaborations and multidisciplinary care.

In the aspect of issues with the teaching and learning of SCD, most respondents felt that they lacked appropriate staff when it came to numbers and training to teach the curriculum.

“Academic staff: Our SCD clinic operates as a multidisciplinary service. At present, there is no dedicated specialists in this field. Consequently, both academic staff and undergraduate/postgraduate (UG/PG) students rotate through the clinic, which may result in a lack of continuity in workflow.” (Southeast Asia respondent)

Respondents also noted lack of designated teaching hours and clinical placements.

“(Lack of) clinical hands-on placements; only clinical observerships available.” (Pacific respondent)

## Discussion

The findings of this study suggest that teaching of SCD across the Asia-Pacific region was active, and that adoption or cross-mapping of the iADH guidance - although varied - was mostly sufficiently fulfilled. This could be considered successful especially since the guidance was developed in 2014. Trends in the sufficiency of fulfilment of this guidance were observed between sub-regions of Asia-Pacific, notably in the Pacific countries that had higher scores across all domains. This may be due to the variation in economic development, population demographics, and the availability of local SCD expertise to champion the teaching of the subject. In Indonesia and Philippines, the area of SCD is considered ’subspecialty’ to Paediatric Dentistry. Under the parent specialty, while geriatric dentistry component is included in SCD's scope, this is focused to a smaller extent. This may be a consequence of population factors, for example Philippines has under 14% of its population aged 65 and above and is not considered an ageing country. However, these findings should be interpreted as exploratory and descriptive, given the uneven regional representation of participating institutions.

Having SCD formally recognised as a specialisation appears to also influence the fulfilment of domains in the guidance. However, the absence of a formal speciality recognition does not necessitate the lack of local SCD training or expertise. Taiwan and South Korea are known to have established SCD education in their dental schools for multiple decades. However, due to national and systemic characteristics, SCD has not been recognised as a dental specialty at their national level, although there are established postgraduate programmes and career pathways for dentists in this field (7). Additionally, dental schools may have unique arrangements within their programmes which results in SCD not being taught. For example, Brunei supports a ‘twinning programme’ where local dental students undergo 3 years of didactic learning before transferring to Dundee, Glasgow, or Newcastle. These dental schools in the United Kingdom would include SCD but were not included in our survey.

Dedicated time and a distinct independent module for SCD may not always have direct benefit on students’ learning outcomes. Dental undergraduate curricula are often crowded with competing subjects and disciplines. Furthermore, while faculty members may view a subject as either independent or embedded, students are faced with a daily cacophony of lectures, tutorials, and clinical placements that rarely align under the same subject or learning objective. Nevertheless, it remains critical for components of SCD to be sufficiently taught, whether independent or embedded, and students’ appropriately assessed on their knowledge, skills, and attitudes. This invariably means support from faculty leadership, funding for expertise and resources required to teach the various topics, and if possible, dedicated clinical time for hands-on management of patients to apply the knowledge gained.

In the training of undergraduates to manage older adult patients, Brandt et al. suggests a concept of “willingness to treat” that encompasses knowledge on ageing, attitudes towards older patients, perceived competence, and empathy (22). The same may be said when broadened to SCD education, where an overarching construct of “*willingness to care*” will encompass the knowledge, perceived skills, attitudes and empathy towards patients requiring special care. Intentional design of SCD curricula, including didactics and practical components, can have significant impact on one's confidence to manage this group of patients (23). Clinical placements, or at least some form of clinical exposure, can help students progress from “conscious incompetence” as result of didactic-focused methodologies to “conscious competence” in more authentic clinical learning environments (24). Beyond the undergraduate curricula, continuous education courses should also be considered, having an impact on one's confidence to manage patients with special care needs (25). Establishing a network between general dentists and SCD specialists can further influence their willingness to manage patients requiring special care (26).

Ultimately, the iADH guidance is invaluable in providing a broad approach to undergraduate SCD teaching. There remains the need for each undergraduate programme to develop fit-for-purpose curricula that train clinicians that can readily meet the needs of the local population and be competent in providing basic oral health care to those requiring special care dentistry.

### Limitations

This study is not without limitations. The convenience and snowball sampling methods gave rise to an imbalanced representation in Asia-Pacific regions, such as with a high response rate from Australia (7 out of 9 dental schools) and no responses from major regions including mainland China and South Asia. Nonetheless, the findings serve as critical pilot data for guidelines review within the limited timeframe. Given that the total number of dental schools in the Asia-Pacific region is substantially larger, the present sample should be interpreted as a subset rather than representative of the region as a whole. Consequently, the findings may have limited generalisabilty and should be considered exploratory, There may also be considerable variation in delivery of SCD content, particularly due to overlapping topics with dental specialties such as medical complexities within oral medicine, or intellectual disability within paediatric dentistry, or geriatric considerations within prosthodontics. The delineation of module content is understandably imprecise, although there are consistent themes drawn from the findings enabling fulfilment of the study's aims.

There may also be variability in how respondents interpret the rating scale domains, particularly across different cultural, institutional, and educational contexts within the Asia-Pacific region. Additionally, respondents may differ in how they interpret the fulfilment of specific domains. For example, there might not be SCD clinical hours or observerships within the overall curricula, but respondents may indicate substantial fulfilment of Domain 6 “*Clinical Management of patients requiring Special Care Dentistry*” made possible through case discussions during lectures or tutorials.

This survey ultimately reports adoption and considerations of its respondents. It does not report on the success of the iADH guidelines, which “*is designed to provide undergraduates with theoretical knowledge and clinical experience and to build skills, positive attitudes and behaviours desirable in SCD*” (11).

### Future directions

While the iADH undergraduate curriculum is adopted widely, there are variations in implementations and assessment within the represented schools, as with other regions of the world (20). Additionally, this study evaluated outcomes of each programme's curriculum mostly at Level 1 (reaction) or 2 (learning) of Kirkpatrick's Four Levels of evaluating training programmes (21), whereas Levels 3 (behaviour) or 4 (results) are more dynamic and complex to evaluate since they would require prolonged observation of the learner's participation and clinical management of patients requiring special care. Future studies can pre-set indicators of students’ learning outcomes over time, and preferably beyond graduation. This allows for a more authentic evaluation of the SCD teaching and provides valuable insight for local reviews of the curriculum. This study also recommends key considerations for the review of the current iADH undergraduate guidance, distilled from the findings of this study:

1. Include a list of topics to enable breadth of content within SCD curriculum
2. Emphasise importance of continual learning of SCD after graduation, given limitation of implementation within undergraduate curricula
3. Implement key indicators to facilitate standardised audits/evaluation of SCD curriculum across schools

## Conclusions

The findings suggest that the iADH undergraduate curriculum guidance is generally well adopted, and many dental schools in Asia-Pacific have incorporated components of SCD, regardless of official state-level recognition of this dental specialty. Often, dental schools experience varying limitations, from resources, manpower, operations, or other systemic factors. Overall, it is crucial that individual dental schools and their SCD curriculum designer(s) fundamentally understand the role of graduated dentists - unique to their specific population needs - to ensure practice-ready clinicians.

## Data Availability

The datasets presented in this article are not readily available because the dataset is available upon reasonable request. Requests to access the datasets should be directed to dengabriellee@nus.edu.sg.
